# Removal of pacemaker leads in extracorporeal circulation

**DOI:** 10.1186/1749-8090-8-S1-P155

**Published:** 2013-09-11

**Authors:** E Likaj, A Kacani, S Dumani, D Hasi, A Alia, A Kenga, L Dibra, A Refatllari

**Affiliations:** 1 Cardiovascular Surgery Department, University Hospital Centre “Mother Theresa”, Tirana, Albania

## Background

The value of extraction of infected or hazardous endocardial pacemakers leads is well established. This review describes our experience with safe and open technique.

## Methods

We reviewed our registry in a period of 20 years (1992 – 2012). We found four patients with pacemaker leads removal in median sternotomy and extracorporeal circulation.

## Results

Overall, 6 leads were removed from 4 patients. The commonest indication for extraction was infection (endocarditis) in three patients (75 %). The other patient was operated because of massive thrombus on the leads. Removal was complete and safe for all the patients. All the patients received epicardial lead with pacemaker pocket located in the rectus abdominis muscle. The postoperative period was free of complications in all the patients.

## Conclusions

Pacemaker infections generally respond to antibiotics and complete hardware removal. However, these principles cannot always be invoked, and the risk of complications is likely to increase when hardware cannot be completely removed. Open lead extraction is an aggressive but safe technique in cases of infected patients with endocarditis unresponsive to antibiotic therapy.

